# Regenerative potential of extracellular vesicles from endometrial mesenchymal stem cells for modulating fibrosis and wound healing

**DOI:** 10.1186/s13287-026-05189-w

**Published:** 2026-07-18

**Authors:** Fang-Yu Lin, Xiao-Tong Tan, Chih-Ming Pan, Chien-Chu Huang, Wan-Ling Chiang, Che-Kai Chang, Wu-Chou Lin, Chiung-Yi Yeh, Yu-Chuan Lin, Ting-Hsun Lin, Der-Yang Cho, Shao-Chih Chiu

**Affiliations:** 1 Graduate Institute of Biomedical Sciences, China Medical University, Taichung, Taiwan, ROC; 2 Translational Cell Therapy Center, China Medical University Hospital, Taichung, Taiwan, ROC; 3 Drug Development Center, China Medical University, Taichung, Taiwan, ROC; 4 Department of Neurosurgery, China Medical University Hospital, Taichung, Taiwan, ROC; 5 Department of Obstetrics and Gynecology, China Medical University Hospital, Taichung, Taiwan, ROC; 6 Department of Medicine, School of Medicine, China Medical University Hospital, Taichung, Taiwan, ROC; 7 Center for Immuno-Oncology, National Cancer Institute, National Institutes of Health, Bethesda, MD USA

**Keywords:** eMSC-EV-enriched preparations, Fibrosis, miR-125b-5p, Smad2, Wound healing

## Abstract

**Background:**

Fibrotic scarring resulting from trauma, burns, or surgery affects over 100 million people annually and is associated with functional, aesthetic, and psychological burdens. Current treatments remain inadequate, highlighting the need for novel, effective, and cell-free therapeutic strategies. Extracellular vesicle-enriched preparations derived from human endometrial mesenchymal stem cells (eMSC-EV-enriched preparations) may possess regenerative and anti-fibrotic potential, yet their roles in scar modulation and underlying mechanisms remain unclear.

**Methods:**

eMSCs were isolated from human endometrial biopsies and characterized via flow cytometry and differentiation assays. The eMSC-EV-enriched preparations were obtained from conditioned medium and verified by TEM, NTA, and Western blotting. Functional assays were conducted in NIH3T3 fibroblasts to assess proliferation, migration, and transwell invasion capacity. A full-thickness cutaneous wound model and a bleomycin-induced dermal fibrosis model were used in C57BL/6 mice to evaluate tissue repair and fibrosis attenuation. Histological and molecular analyses were performed to assess collagen deposition, fibrosis-associated markers, and related signaling pathways. The potential involvement of miR-125b-5p in Smad2-related signaling was explored.

**Results:**

The eMSC-EV-enriched preparations displayed characteristic vesicular morphology and marker expression. Compared with BMMSC-EV-enriched preparations, eMSC-EV-enriched preparations more effectively reduced fibroblast proliferation and migration, with decreased transwell invasion capacity in vitro. In vivo, the eMSC-EV-enriched preparations enhanced wound closure, reduced collagen deposition, and were associated with improved collagen organization and an increased number of appendage-like structures. Expression of α-SMA and collagen I and III was reduced in tissues treated with the eMSC-EV-enriched preparations. Furthermore, the preparations were associated with increased miR-125b-5p levels and decreased Smad2/p-Smad2 expression, suggesting involvement of the miR-125b-5p/Smad2 axis. In the bleomycin model, the eMSC-EV-enriched preparations attenuated dermal thickening and collagen accumulation during fibrosis induction.

**Conclusions:**

Our findings indicate that eMSC-EV-enriched preparations improve repair quality and attenuate fibrosis development, potentially through modulation of fibroblast activity and involvement of the miR-125b-5p/Smad2 signaling pathway. These findings support further investigation of eMSC-EV-enriched preparations as a cell-free strategy for improving tissue remodeling in fibrotic skin conditions.

**Supplementary Information:**

The online version contains supplementary material available at https://doi.org/10.1186/s13287-026-05189-w.

## Background

Each year, approximately 100 million individuals develop scars resulting from trauma, burns, surgery, infections, or chronic diseases [1]. Fibrotic scars, including hypertrophic scars, are characterized by excessive deposition of collagen and extracellular matrix (ECM) components, loss of cutaneous appendages, and disorganized tissue architecture [2, 3]. These scars not only lead to physical symptoms such as pain, pruritus, and limited mobility but also impose significant aesthetic, psychological, and social burdens [4]. Despite decades of research, effective therapeutic strategies for preventing or reversing scarring remain elusive [5]. Achieving scar-free skin regeneration, therefore, remains a critical yet unmet clinical goal [6], underscoring the urgent need for more targeted and effective anti-scarring therapies.

Stem cell therapy has been extensively explored for its potential in wound healing due to the regenerative capabilities of mesenchymal stem cells (MSCs) [7, 8]. However, traditional MSC-based therapies face several challenges, including high costs, limited durability, batch variability, and potential tumorigenicity [9, 10]. Notably, the therapeutic effects of MSCs are mediated primarily through paracrine signaling rather than direct differentiation [11–13]. Among the key paracrine mediators, MSC-derived extracellular vesicles (MSC-EVs) have gained considerable attention as promising cell-free alternatives, capable of delivering functional cargo such as DNA, proteins, mRNAs, and microRNAs to recipient cells [14–18]. These nano-sized vesicles (50–200 nm) recapitulate many of the regenerative and immunomodulatory properties of their parent cells [19], and have demonstrated superior efficacy across all phases of wound healing compared to whole-cell MSC therapies [20].

The human endometrium is a uniquely regenerative tissue that undergoes over 400 cycles of scar-free remodeling during a woman’s reproductive lifespan, largely attributed to endometrial mesenchymal stem cells (eMSCs) [21]. In vitro, eMSCs closely resemble bone marrow-derived MSCs (BMMSCs), expressing classical MSC markers along with specific surface markers such as SUSD2, CD140b, and CD146 [22–24]. These markers are associated with the high proliferative capacity and multipotency of eMSCs [25, 26]. Importantly, eMSCs can be harvested via a minimally invasive endometrial biopsy without the need for anesthesia, offering a practical and less invasive alternative to other MSC sources. eMSCs have demonstrated regenerative and anti-fibrotic properties through paracrine mechanisms that promote angiogenesis, modulate inflammation, and support tissue remodeling [27–30].

Extracellular vesicle-enriched preparations derived from endometrial mesenchymal stem cells (eMSC-EV-enriched preparations) have recently emerged as a promising cell-free therapeutic modality. This potential is supported by the intrinsic regenerative capacity of the endometrium, which undergoes cyclical, scar-free remodeling during menstruation and postpartum recovery. Beyond their physiological role, eMSC-EV-enriched preparations have shown therapeutic efficacy in various preclinical models of fibrotic diseases. In myocardial infarction models, they enhanced cardiac function, promoted angiogenesis, reduced fibrosis, and attenuated ventricular remodeling [31, 32]. eMSC-EV-enriched preparations have also been reported to ameliorate pulmonary fibrosis [33, 34], further underscoring their broad anti-fibrotic potential. A more critical consideration is that the underlying mechanisms of endometrial fibrosis resemble those of cutaneous wound healing and scarring [35]. These findings suggest that eMSC-EV-enriched preparations possess regenerative and fibrosis-modulating properties that may be applicable to fibrotic wound healing. However, their therapeutic efficacy and underlying mechanisms in cutaneous scar fibrosis remain largely uncharacterized. Moreover, comparative analyses of EV-enriched preparations from other MSC sources, such as BMMSCs, remain limited.

In this study, we comprehensively evaluated the effects of eMSC-EV-enriched preparations on cutaneous wound healing, fibroblast behavior, and scar formation. Our results indicate that eMSC-EV-enriched preparations enhance wound closure and attenuate fibrosis development, potentially through modulation of the miR-125b-5p/Smad2 signaling axis. These findings provide evidence supporting the therapeutic potential of eMSC-EV-enriched preparations as a cell-free strategy for improving fibrotic wound repair.

## Methods

### Human endometrial tissue collection

Human endometrial tissue samples were obtained from five healthy female volunteers (mean age 44.8 ± 4.5 years) at China Medical University Hospital (CMUH), Taichung, Taiwan. All participants were recruited from individuals scheduled for non-pathological gynecological assessments or procedures. Endometrial tissues were collected via dilation and curettage performed during routine clinical care. Eligible participants were premenopausal and in good general health. Exclusion criteria included: (1) pregnancy or breastfeeding; (2) presence of radioactive implants; (3) fever or acute infections; (4) active collagen vascular diseases; (5) acute cardiovascular diseases; (6) acute viral hepatitis; (7) syphilis; (8) HIV infection; (9) individuals deemed unsuitable for participation by the attending physician; and (10) postmenopausal status. The study protocol was reviewed and approved by the Institutional Review Board (IRB) of CMUH (Approval No. CMUH113-REC2-156, effective January 25, 2025). All participants provided written informed consent after being fully informed of the study’s purpose and procedures. All tissue samples were used exclusively for research purposes, in accordance with institutional and ethical guidelines.

### Isolation and culture of human endometrial mesenchymal stem cells (eMSCs)

Human endometrial mesenchymal stem cells (eMSCs) were isolated and cultured as previously described [36]. Briefly, freshly collected endometrial tissue specimens were placed in Hank’s Balanced Salt Solution (HBSS; Invitrogen, Carlsbad, CA, USA) supplemented with 1% (v/v) Penicillin-Streptomycin (Pen/Strep; Sigma, USA) and washed three times with the same solution. Tissues were enzymatically digested in HBSS containing 1 mg/mL collagenase type I (Sigma, USA) at 37 °C in a humidified atmosphere of 5% CO_2_ for 60 min. The digestion was terminated by adding growth medium composed of DMEM/F-12 (Gibco, USA) supplemented with 10% FBS and 1% Pen/Strep. To eliminate glandular epithelial cells, the resulting cell suspension was sequentially filtered through 70 μm and then 40 μm Falcon cell strainers. The filtered cells were centrifuged at 300 × *g* for 10 min, and the cell pellet was resuspended in growth medium. Cells were seeded into 10-cm culture dishes containing 10 mL of DMEM/F-12 with 10% FBS and 1% Pen/Strep and maintained at 37 °C in a humidified incubator with 5% CO_2_. The culture medium was changed every three days until the cells reached approximately 80–90% confluency. eMSCs between passages 5 and 10 were used for all subsequent experiments.

### Characterization and differentiation of eMSCs

The immunophenotypic profile of eMSCs was assessed by flow cytometry. Cells were incubated with fluorescently conjugated monoclonal antibodies against CD34, CD45, CD73, CD90, CD105, SUSD2, CD146, and CD140b (all from BioLegend, California, USA), and analyzed using a spectral flow cytometer (SONY SA3800, Japan). To evaluate the multipotent differentiation potential of eMSCs, cells were subjected to adipogenic, osteogenic, and chondrogenic differentiation using the Human Mesenchymal Stem Cell Functional Identification Kit (R&D Systems, MN, USA), according to the manufacturer’s instructions. During the induction period, the medium was refreshed every 3–4 days. After 21 days of differentiation, cells were fixed with 4% paraformaldehyde and stained with lineage-specific dyes to confirm successful differentiation: Oil Red O (Sigma, St. Louis, USA) for intracellular lipid accumulation (adipogenesis), Alizarin Red S (Sigma, Darmstadt, Germany) for calcium deposition (osteogenesis), and Alcian Blue (Sigma, St. Louis, USA) for sulfated proteoglycan production (chondrogenesis). Stained cells were visualized and imaged using an inverted phase-contrast microscope.

### Isolation and characterization of extracellular vesicle-enriched preparations

Extracellular vesicle-enriched preparations (EV-enriched preparations) were generated from endometrial mesenchymal stem cells (eMSCs) and bone marrow-derived mesenchymal stem cells (BMMSCs; Evercyte, Cat# CHT-063-0292, Vienna, Austria) using an identical conditioning and concentration workflow to ensure comparability in head-to-head analyses. Both cell types were cultured to approximately 90% confluency and subjected to a harmonized serum-free conditioning period (12–24 h). Conditioned media were collected and centrifuged at 300 × *g* for 5 min to remove cells and debris, followed by filtration and concentration using centrifugal filter units (Pall Corp., Puerto Rico, USA) at 4,000 × *g* for 1 h to obtain EV-enriched preparations, as previously described [37, 38]. In both eMSCs and BMMSCs, cell viability after serum-free conditioning exceeded 95% as assessed by trypan blue exclusion. Morphology was evaluated by transmission electron microscopy (TEM). Particle size distribution and concentration were determined using nanoparticle tracking analysis (NTA; ZetaView system). Particle-to-protein ratios were calculated using NTA particle counts and total protein concentrations quantified by the BCA assay, and were comparable between eMSC- and BMMSC-EV-enriched preparations. For downstream functional assays, EV-enriched preparations were normalized based on equal total protein amounts to ensure comparability between sources. The expression of canonical EV surface markers CD9, CD63, and CD81 was confirmed by Western blot analysis.

### Uptake assay of EV-enriched preparations

For uptake analysis, EV-enriched preparations derived from eMSCs and BMMSCs were labeled with the green fluorescent dye PKH67 (Sigma, St. Louis, USA) under identical conditions. Briefly, EV-enriched preparations were resuspended in Diluent C to prevent dye aggregation. PKH67 dye was diluted in Diluent C to obtain a working solution, and 250 µL of EV-enriched preparations were incubated with dye at a final concentration of 1 × 10^− 6^ M for 5 min at room temperature. The labeling reaction was terminated by adding an equal volume of EV-depleted FBS to bind excess dye. To remove unincorporated dye, labeled EV-enriched preparations were washed three times using 100 kDa molecular weight cut-off centrifugal filter units at 4,000 × *g* for 15 min per wash. Dye-only controls (PKH67 processed in parallel in the absence of EV-enriched preparations) were subjected to the same washing procedure to assess background fluorescence. Total protein concentration of labeled EV-enriched preparations was quantified using the Bradford assay to ensure equal dosing between groups. Labeled EV-enriched preparations and dye-only controls were added to fibroblast cultures in serum-free medium and incubated for 24 h. Cells were fixed with 4% paraformaldehyde, washed three times with PBS, and nuclei were counterstained with DAPI. Fluorescence images were acquired using a confocal spectral microscope (Leica TCS SP8X) under identical acquisition settings for all groups.

### Culture of NIH3T3 mouse fibroblast cell line

The NIH3T3 mouse fibroblast cell line (ATCC, Manassas, VA, USA) was cultured in DMEM (Thermo Fisher Scientific, Waltham, MA, USA) supplemented with 10% FBS and 1% Pen/Strep. Cells were maintained at 37 °C in a humidified incubator with 5% CO_2_ and passaged when they reached 80–90% confluency.

### Cell proliferation assay

Fibroblast cells were seeded in a CytoView-Z™ 96-well plate (Axion BioSystems, USA) at a density of 5,000 cells per well in 100 µL of complete culture medium. After cell attachment, the medium was replaced with serum-free medium supplemented with 150 µg of eMSC- or BMMSC-EV-enriched preparations. Real-time monitoring of cell proliferation was conducted for 24 h using the MaestroZ™ impedance-based system (Axion BioSystems, USA). Changes in electrical impedance, which correlate with cell number and adhesion, were recorded and analyzed as indicators of proliferative activity.

### Migration and invasion assays

Fibroblast migration and invasion were evaluated using a modified Boyden chamber assay (Costar, Cambridge, MA, USA). For the migration assay, 1 × 10^5^ fibroblast cells suspended in serum-free medium containing 150 µg of eMSC- or BMMSC-EV-enriched preparations were seeded into the upper chamber of transwell inserts (8 μm pore size). For the invasion assay, the upper chambers were pre-coated with Matrigel (BD Biosciences, Franklin Lakes, NJ, USA). The lower chambers were filled with complete medium containing serum as a chemoattractant. After incubation for 24 h at 37 °C, non-migrated or non-invaded cells remaining on the upper surface of the membrane were gently removed using a cotton swab. The inserts were then washed with PBS, fixed in 4% paraformaldehyde, and stained with 0.5% crystal violet. Cells that had migrated or invaded onto the lower surface of the membrane were visualized using an inverted microscope, and images were captured from five randomly selected fields per insert. The number of stained cells was quantified using ImageJ software.

### Western blotting

Fibroblast cells were washed twice with ice-cold PBS and lysed in RIPA buffer (Invitrogen, USA) supplemented with a protease inhibitor cocktail (Roche, Switzerland). Lysates were incubated on ice for 30 min and centrifuged at 12,000 rpm for 20 min at 4 °C to remove cellular debris. The protein concentration of the supernatants was determined using a BCA Protein Assay Kit (Thermo Fisher Scientific, USA). Equal amounts of total protein (30 µg) were separated by 10% SDS-polyacrylamide gel electrophoresis (SDS-PAGE) and transferred onto PVDF membranes (Millipore, Bedford, MA, USA). Membranes were blocked with 5% non-fat milk in TBST (Tris-buffered saline containing 0.1% Tween-20) for 1 h at room temperature and then incubated overnight at 4 °C with the following primary antibodies: anti-α-SMA (1:1000, GeneTex), anti-Smad2 (1:1000, GeneTex), anti-phospho-Smad2 (p-Smad2; 1:1000, GeneTex), and anti-GAPDH (1:1000, Servicebio). After washing three times with TBST, membranes were incubated with horseradish peroxidase (HRP)-conjugated secondary antibodies for 1 h at room temperature. Protein bands were visualized using enhanced chemiluminescence (ECL) reagents (Millipore, USA) and imaged with the ChemiDoc Imaging System (Bio-Rad, Hercules, CA, USA).

### Quantitative real-time PCR (qRT-PCR)

Skin wound tissues were harvested after treatment to evaluate the mRNA expression levels of genes associated with wound healing. Total RNA was isolated using the RNeasy Mini Kit (Qiagen) following the manufacturer’s protocol. The concentration and purity of RNA were assessed using a spectrophotometer, and equal amounts of RNA were used for complementary DNA (cDNA) synthesis. Reverse transcription was carried out using the High-Capacity cDNA Reverse Transcription Kit (Applied Biosystems) according to the manufacturer’s instructions. Quantitative real-time PCR was conducted using SYBR Green PCR Master Mix (Applied Biosystems) on an ABI StepOnePlus Real-Time PCR System (Applied Biosystems). The thermal cycling conditions were as follows: an initial denaturation at 95 °C for 10 min, followed by 40 amplification cycles of 15 s at 95 °C and 1 min at 60 °C. Gene expression levels were quantified using the comparative CT (ΔΔCt) method, and *GAPDH* was used as the internal reference gene. All reactions were performed in triplicate, and results were expressed as fold change relative to the control group.

### Animal model of cutaneous wound healing

Female C57BL/6 mice (6–8 weeks old) were obtained from the National Laboratory Animal Center (Taiwan) and maintained under standard conditions (20–24 °C, 45–65% relative humidity) with a 12-h light-dark cycle. All animal experiments were approved by the Institutional Animal Care and Use Committee (IACUC) of China Medical University. The work has been reported in line with the ARRIVE guidelines 2.0. Before surgery, mice were briefly anesthetized with 2% isoflurane via inhalation. Euthanasia was performed by CO_2_ inhalation in accordance with institutional guidelines. To evaluate the therapeutic effects of different treatments on wound healing, mice were randomly assigned to one of four groups (*n* = 5 per group): PBS, antibiotic ointment (containing neomycin), BMMSC-EV-enriched preparations, or eMSC-EV-enriched preparations. Under isoflurane anesthesia, a 1 × 1 cm^2^ full-thickness excisional wound was created on the dorsal skin of each mouse. Beginning on the day following surgery, mice in the eMSC-EV-enriched preparations and BMMSC-EV-enriched preparations groups received subcutaneous injections of EV-enriched preparations (150 µg in 150 µL PBS) around the wound margins, administered twice per week for 2 weeks. The PBS group received an equal volume of PBS on the same schedule, while the antibiotic group was treated with topical neomycin-containing ointment applied directly to the wound surface at the same time points. Wound images were taken on days 0, 3, 7, 10, and 14 to assess wound closure. At the end of the study period, mice were euthanized, and wound tissues were collected for histological and molecular analysis.

### Histological and immunohistochemical analysis

Wound tissues were harvested at the experimental endpoint, fixed in 4% paraformaldehyde (pH 7.4), dehydrated through graded ethanol, and embedded in paraffin. Paraffin blocks were sectioned at 3 μm thickness and stained with hematoxylin and eosin (H&E), Masson’s trichrome, and Sirius Red to evaluate tissue morphology and collagen deposition. For each section, five randomly selected high-power fields (HPFs, 20×) were analyzed in a blinded manner. Cutaneous appendages were manually counted, and Sirius Red-stained sections were imaged under brightfield to quantify total collagen content as the percentage area of positive staining within defined regions of interest (ROIs) using ImageJ. For immunohistochemistry (IHC), sections underwent antigen retrieval, followed by incubation with 3% hydrogen peroxide for 20 min to block endogenous peroxidase activity. After blocking with 5% bovine serum albumin (BSA) in PBS, sections were incubated overnight at 4 °C with primary antibodies against CD31 (1:100, GeneTex), Ki67 (1:200, Servicebio), α-smooth muscle actin (α-SMA, 1:500, GeneTex), and Smad2 (1:500, GeneTex). The next day, sections were washed and incubated with biotin-conjugated secondary antibodies, followed by a polymer-based detection system and diaminobenzidine (DAB) chromogenic development. Positively stained cells or areas were quantified using ImageJ to evaluate marker expression across different treatment groups.

### Cell transfection of miR-125b-5p

Fibroblast cells were seeded in 12-well plates and transfected with 100 nM of synthetic miR-125b-5p, scramble control, or a non-targeting negative control RNA using the TransIT-X2^®^ Dynamic Delivery System (Mirus Bio, Madison, WI, USA), following the manufacturer’s protocol. All transfection experiments were conducted in triplicate to ensure reproducibility. At 48 h post-transfection, cells were harvested for downstream analyses. Cell proliferation was evaluated, and protein expression levels were determined by Western blotting. To assess the fibrotic response, the expression of Smad2 and phosphorylated Smad2 (p-Smad2) was specifically examined.

### Bleomycin-induced dermal fibrosis model with co-administration of eMSC-EV-enriched preparations

C57BL/6 mice (6–8 weeks old) were randomly assigned to two groups (*n* = 5 per group): bleomycin alone or bleomycin with eMSC-EV-enriched preparations. Localized dermal fibrosis was induced by intradermal injection of bleomycin (100 µL of 1 mg/mL solution) into the dorsal skin every other day for 4 weeks. Mice in the eMSC-EV-enriched preparations group received intradermal co-administration of eMSC-EV-enriched preparations (150 µg in 150 µL PBS) at the same site and schedule, while mice in the bleomycin-alone group received an equal volume of PBS. All histological analyses were performed by investigators blinded to group allocation. At the end of the 4-week period, mice were euthanized, and dorsal skin tissues were collected for histology (H&E, Masson’s trichrome, and Sirius Red) to assess dermal thickness, collagen deposition, and fibrotic changes.

### Statistical analysis

All experiments were performed with at least three independent replicates. Data are presented as the mean ± standard error of the mean (SEM). Statistical analyses were conducted using a Student’s *t*-test or paired *t*-test for comparisons between two groups. For animal experiments involving more than two groups, one-way analysis of variance (ANOVA) was used. A *p*-value of less than 0.05 was considered statistically significant.

## Results

### Characterization of eMSCs and eMSC-EV-enriched preparations

**Fig. 1:**
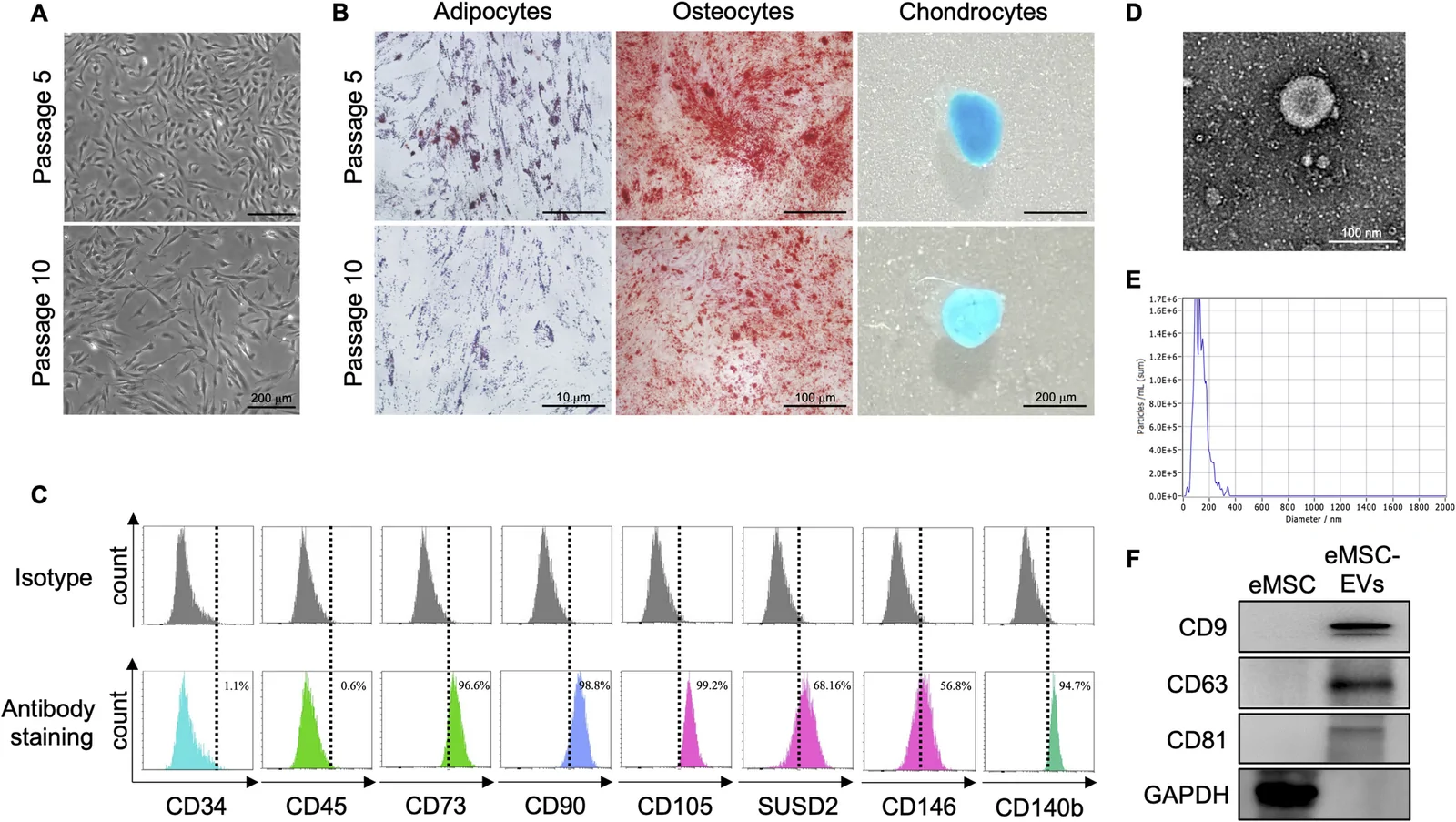
The identification of eMSCs and eMSC-EV-enriched preparations. **A** The morphology of eMSCs at passages 5 and 10 observed by inverted microscopy (scale bar = 200 μm). **B** Adipogenic, osteogenic, and chondrogenic differentiation were assessed using Oil Red O staining (scale bar = 10 μm), Alizarin Red S staining (scale bar = 100 μm), and Alcian Blue staining (scale bar = 200 μm), respectively. **C** Flow cytometry analysis of eMSCs at passage 10 showing the expression of hematopoietic stem cell markers (CD34: 1.1%, CD45: 0.6%), mesenchymal stem cell markers (CD73: 96.6%, CD90: 98.8%, and CD105: 99.2%), and endometrial stem cell markers (SUSD2: 68.16%, CD146: 56.8%, CD140b: 94.7%). **D** The morphology of eMSC-EV-enriched preparations detected by TEM (transmission electron microscopy) (scale bar = 100 nm). **E** Particle size distribution of eMSC-EV-enriched preparations measured by NTA (nanoparticle tracking analysis). **F** Immunoblot analysis of extracellular vesicle markers (CD9, CD63, and CD81) (Full-length blots are presented in Supplementary Fig. 1). eMSC-EVs in the figures represent eMSC-EV-enriched preparations

Primary endometrial mesenchymal stem cells (eMSCs) were isolated from human endometrial tissue and exhibited a spindle-shaped morphology at both passages 5 and 10 (Fig. 1A). Multipotency was confirmed by adipogenic, osteogenic, and chondrogenic differentiation assays performed at passages 5 and 10. Oil Red O staining showed lipid droplets, Alizarin Red S staining revealed calcium deposition, and Alcian Blue staining detected acid mucopolysaccharides. These findings confirmed the morphology and trilineage differentiation capacity of eMSCs, both of which were preserved at early and late passages (Fig. 1B). Flow cytometry analysis showed that eMSCs were highly positive for MSC (mesenchymal stem cell) markers CD73, CD90, and CD105, while negative for HSC (hematopoietic stem cell) markers CD34 and CD45. In addition, eMSCs exhibited positive expression of SUSD2, CD146, and CD140b, which are characteristic markers of endometrial stem cells (Fig. 1C). Flow cytometric signals were analyzed relative to isotype controls. A high expression of stem cell markers and strong differentiation potential confirmed the quality of eMSCs at both early and late passages. eMSCs at passages 5–10 were used for subsequent experiments. Furthermore, eMSC-EV-enriched preparations were subsequently collected from serum-free conditioned medium of eMSCs and displayed a cup-shaped morphology in the TEM image (Fig. 1D). NTA analysis revealed vesicles with a diameter range of 50–200 nm and a concentration of 1 × 10^9^ particles/mL (Fig. 1E). Immunoblotting confirmed the presence of EV-associated markers CD9, CD63, and CD81 (Fig. 1F). Collectively, these results indicate that the eMSC-EV-enriched preparations display characteristic morphological, size, and protein features consistent with extracellular vesicles.

### eMSC-EV-enriched preparations modulated fibroblast proliferation, migration, and matrix-interactive behavior in vitro

**Fig. 2:**
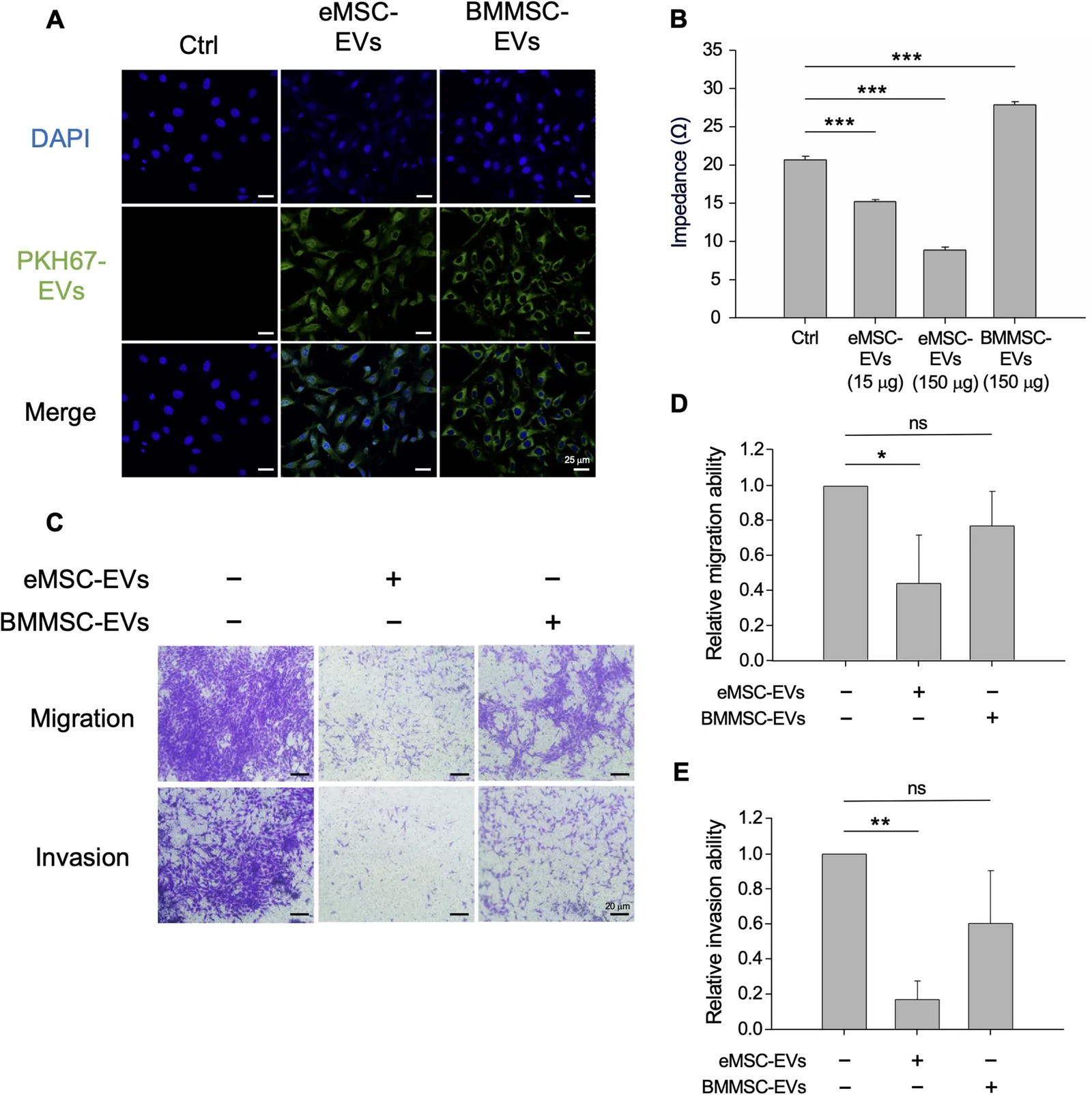
eMSC-EV-enriched preparations modulate fibroblast proliferation, migration, and invasion in vitro. **A** Immunofluorescence staining of PKH67-labeled eMSC- or BMMSC-EV-enriched preparations internalized by fibroblasts; blue: DAPI, green: PKH67-labeled EV-enriched preparations (scale bar = 25 μm). **B** The effects of eMSC- or BMMSC-EV-enriched preparations on fibroblast proliferation for 24 h were detected by the MaestroZ real-time cell analyzer. **C**–**E** The effects of eMSC- or BMMSC-EV-enriched preparations on fibroblast migration and invasion were evaluated by transwell assay at 24 h (scale bar = 20 μm). The data are shown as mean ± SEM (*p < 0.05, **p < 0.01, ***p < 0.001, ns: no difference). eMSC-EVs and BMMSC-EVs in the figures represent eMSC- and BMMSC-EV-enriched preparations, respectively

Pathological scarring often arises from delayed wound healing and is characterized by excessive proliferation of fibroblasts [39, 40]. Previous studies have demonstrated that eMSCs possess in vitro properties comparable to those of BMMSCs [22–24]. Notably, BMMSC-EV-enriched preparations have been shown to effectively promote cutaneous wound healing [41]. To investigate the distinct effects of EV-enriched preparations derived from different MSC sources on fibroblast behavior, we performed a comparative in vitro analysis of eMSC-EV-enriched preparations and BMMSC-EV-enriched preparations. Fibroblasts were treated with eMSC- or BMMSC-EV-enriched preparations, and successful internalization of EV-enriched preparations was confirmed by the uptake of PKH67-labeled EV-enriched preparations. Dye-only controls were included and showed no detectable fluorescence (Fig. 2A). Treatment with eMSC-EV-enriched preparations significantly inhibited fibroblast proliferation in a dose-dependent manner after 24 h, whereas BMMSC-EV-enriched preparations enhanced fibroblast proliferation (Fig. 2B, *p* < 0.001). Furthermore, eMSC-EV-enriched preparations markedly suppressed fibroblast migration compared to the control group (Fig. 2C-D, *p* < 0.05). In addition, fibroblast invasion was assessed using a Matrigel-coated transwell system. eMSC-EV-enriched preparations significantly reduced invasion relative to controls, whereas BMMSC-EV-enriched preparations exhibited only a mild inhibitory effect (Fig. 2C-E, *p* < 0.01). This assay serves as a supportive proxy for fibroblast behavior under matrix-like conditions, reflecting aspects of matrix-interactive activity in vitro. Collectively, these findings indicate that eMSC-EV-enriched preparations modulate multiple functional aspects of fibroblast activity in vitro, which may be relevant to fibroblast-mediated tissue remodeling and matrix interactions associated with scarring processes.

### eMSC-EV-enriched preparations enhanced cutaneous wound healing in a murine model compared to BMMSC-EV-enriched preparations

**Fig. 3:**
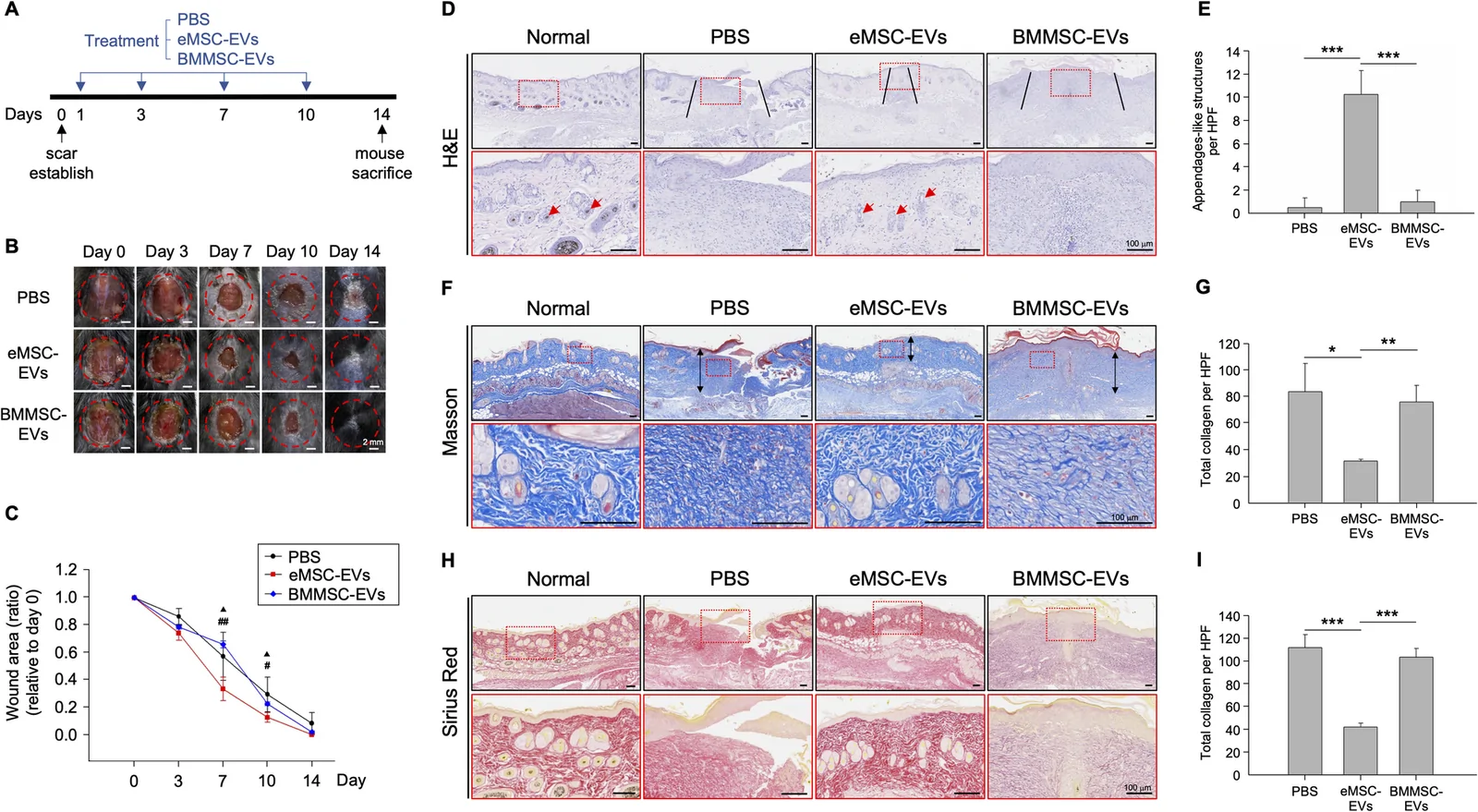
The effects of eMSC-EV-enriched preparations on the excisional wound model of C57BL/6 mice compared to BMMSC-EV-enriched preparations. **A** Schematic diagram illustrating the experimental design. **B** Digital photographs of the wound area during the healing period (scale bar = 2 mm). **C** Quantitative analysis of wound area over time using ImageJ software. **D**, **E** H&E staining images (black line: wound margins; red rectangle: high-power field (HPF); red arrows: appendage-like structures; scale bar = 100 μm) and the count of appendage-like structures in the healed skin. *n* = 5 **F**, **G** Masson’s trichrome staining images showing collagen deposition. (blue color; double arrows: collagen length; scale bar = 100 μm) and quantification of total collagen content. *n* = 5 (H, I) Sirius Red staining of healed skin (scale bar = 100 μm) and quantification of collagen content. *n* = 5. The data are shown as mean ± SEM (*p < 0.05, **p < 0.01, ***p < 0.001; ^▲^*p* < 0.05 compared to the PBS group; ^#^*p* < 0.05, ^##^*p* < 0.01 compared to the BMMSC-EV-enriched preparations group). eMSC-EVs and BMMSC-EVs in the figures represent eMSC- and BMMSC-EV-enriched preparations, respectively

To assess and compare the wound healing efficacy of EV-enriched preparations derived from eMSCs and BMMSCs, a full-thickness excisional wound model was established on the dorsal skin of C57BL/6 mice. Mice were randomly assigned to receive subcutaneous injections of eMSC-EV-enriched preparations, BMMSC-EV-enriched preparations, or an equal volume of PBS as a control (Fig. 3A). A significant reduction in wound area was observed in the eMSC-EV-enriched preparations group on days 7 and 10 compared to both the PBS and BMMSC-EV-enriched preparations groups (Fig. 3B, C, *p* < 0.05 and *p* < 0.01). To further evaluate the quality of tissue regeneration, histological analyses were conducted on wound tissues harvested at day 14 post-surgery, using hematoxylin and eosin (H&E), Masson’s trichrome, and Sirius Red staining. The number of appendage-like structures (hair follicles and sebaceous glands) was quantified from H&E-stained sections of the healed skin. The eMSC-EV-enriched preparations group exhibited reduced wound margins (black line) and a significantly greater number of appendage-like structures (red arrows), compared to both the PBS and BMMSC-EV-enriched preparations groups (Fig. 3D, E, *p* < 0.001). According to previous studies, normal skin exhibits a distinct basket-weave collagen structure, whereas injured skin displays disrupted collagen organization with a parallel arrangement during recovery [42]. Masson’s trichrome staining showed that collagen fibers in the eMSC-EV-enriched preparations group were organized in a basket-weave pattern, resembling normal skin, while those in the PBS and BMMSC-EV-enriched preparations groups showed parallel collagen alignment. In addition, the eMSC-EV-enriched preparations group exhibited significantly reduced and thinner collagen deposition (double arrows, blue color) compared to the other two groups (Fig. 3F, G, *p* < 0.05 and *p* < 0.01). Consistent with these findings, Sirius Red staining revealed overall collagen deposition and fiber organization; the PBS and BMMSC-EV-enriched preparations groups showed higher total collagen accumulation and denser, more parallel-aligned fibers than the eMSC-EV-enriched preparations group (Fig. 3H, I, *p* < 0.001). Collectively, these findings indicate that eMSC-EV-enriched preparations improve histological features and promote the formation of appendage-like structures in healing skin more effectively than BMMSC-EV-enriched preparations, suggesting significant potential for high-quality cutaneous repair.

### eMSC-EV-enriched preparations enhanced wound healing beyond conventional topical therapy

**Fig. 4:**
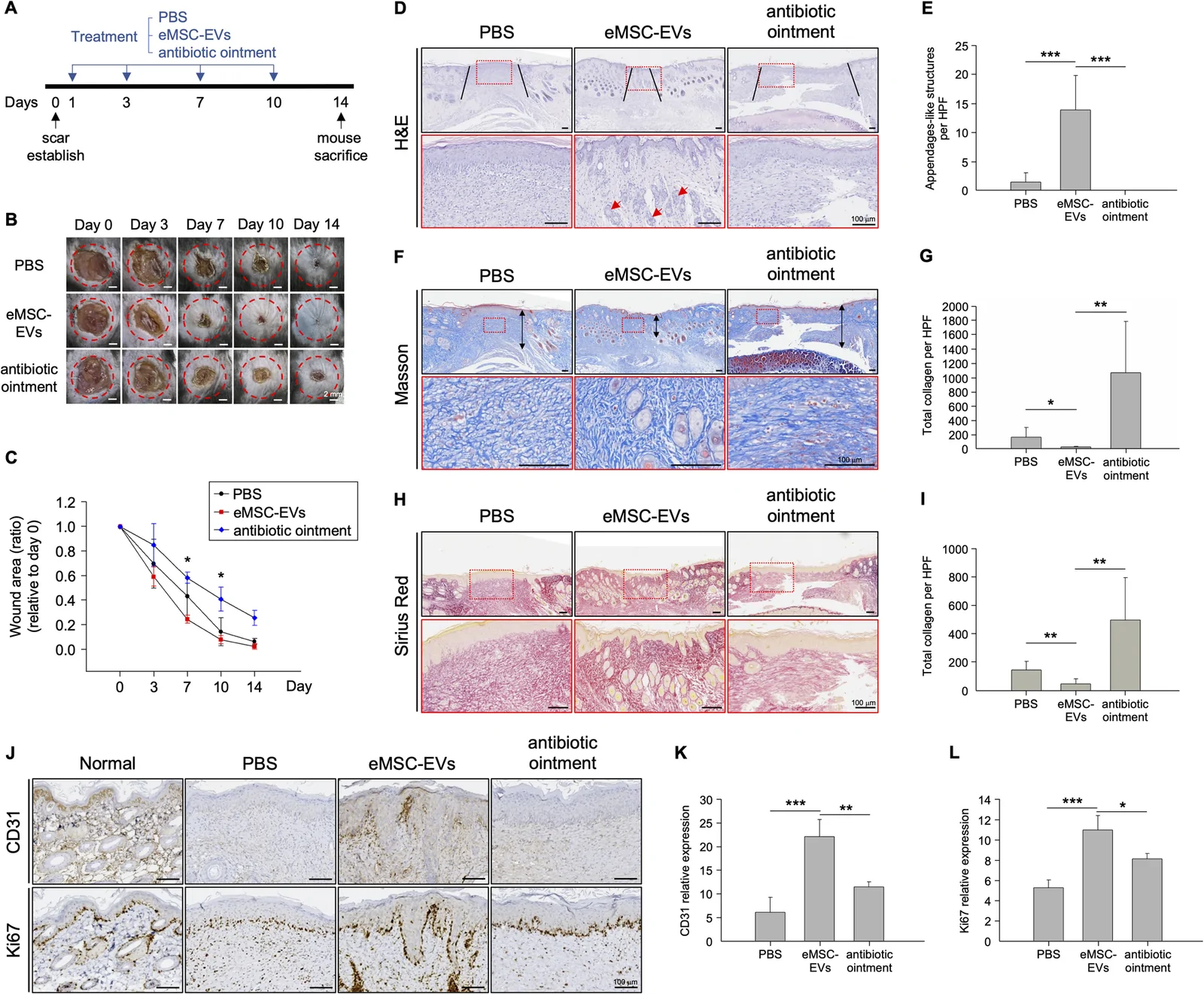
The effects of eMSC-EV-enriched preparations on the excisional wound model of C57BL/6 mice compared to conventional topical therapy. **A** Schematic diagram illustrating the experimental design. **B** Digital photographs of the wound area during the healing period (scale bar = 2 mm). **C** Quantitative analysis of wound area over time using ImageJ software. **D**, **E** H&E staining images (black line: wound margins; red rectangle: high-power field (HPF); red arrows: appendage-like structures; scale bar = 100 μm) and the count of appendage-like structures in the healed skin. *n* = 5 **F**, **G** Masson’s trichrome staining images showing collagen deposition. (blue color; double arrows: collagen length; scale bar = 100 μm) and quantification of total collagen content. *n* = 5 **H**, **I** Sirius Red staining of healed skin (scale bar = 100 μm) and quantification of collagen content. *n* = 5 **J**–**L** CD31 and Ki67 immunohistochemical staining and quantification of the healed skin (scale bar = 100 μm). *n* = 5. The data are shown as mean ± SEM (*p < 0.05, **p < 0.01, ***p < 0.001). eMSC-EVs in the figures represent eMSC-EV-enriched preparations

Topical antibiotic ointments are commonly used in clinical wound care for their ability to prevent bacterial infections, reduce inflammation, and maintain a moist environment conducive to healing [43]. To evaluate the therapeutic potential of eMSC-EV-enriched preparations relative to conventional topical treatments, we conducted a controlled study using a murine wound healing model. Mice were treated with subcutaneous injections of PBS, eMSC-EV-enriched preparations, or a topical antibiotic ointment, which served as the positive control (Fig. 4A). As shown in Fig. 4B and C, treatment with eMSC-EV-enriched preparations significantly reduced the wound area on days 7 and 10 compared to both the PBS and antibiotic ointment groups. Moreover, wounds in the eMSC-EV-enriched preparations group exhibited a smaller area (black line) and a significantly higher number of appendage-like structures (red arrows) compared to the other two groups (Fig. 4D, E, *p* < 0.001). Masson’s trichrome staining revealed a basket-weave collagen structure in the eMSC-EV-enriched preparations group, whereas the PBS and antibiotic ointment groups showed a more disorganized, parallel collagen arrangement. Additionally, total collagen deposition (double arrows, blue color) in the eMSC-EV-enriched preparations group was markedly reduced and thinner compared to both control groups (Fig. 4F, G, *p* < 0.05 and *p* < 0.01). Consistent with these observations, Sirius Red staining showed higher total collagen accumulation and denser, more parallel-aligned fibers in the PBS and antibiotic ointment groups compared to the eMSC-EV-enriched preparations group (Fig. 4H, I, *p* < 0.01). To further evaluate the therapeutic effects on angiogenesis and cell proliferation, we examined CD31 and Ki67 expression, respectively. Immunostaining revealed enhanced capillary density in the eMSC-EV-enriched preparations group, as indicated by increased CD31 expression (Fig. 4J, K, *p* < 0.01 and *p* < 0.001), and elevated Ki67 levels suggested greater cellular proliferation around the wound site (Fig. 4J, L, *p* < 0.05 and *p* < 0.001), compared to both control groups. Overall, these results suggest that eMSC-EV-enriched preparations contribute to improved tissue organization and wound healing, highlighting their potential value for cutaneous repair.

### eMSC-EV-enriched preparations modulate fibroblast activation and ECM-related markers

**Fig. 5:**
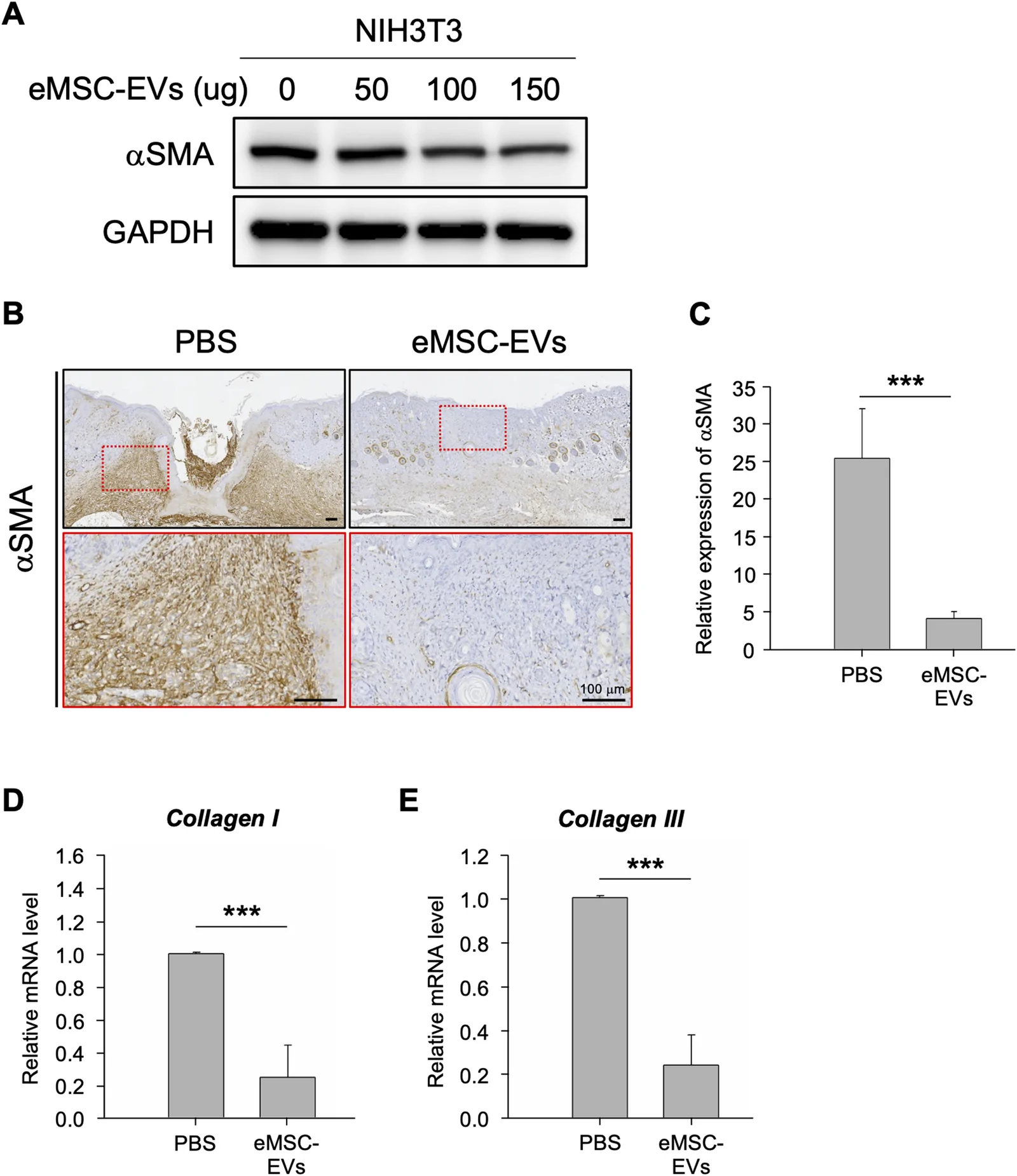
eMSC-EV-enriched preparations modulate fibroblast activation and ECM-associated markers. **A** α-SMA expression in fibroblasts treated with different doses of eMSC-EV-enriched preparations (Full-length blots are presented in Supplementary Fig. 2). **B**, **C** α-SMA immunohistochemistry staining of wound tissue treated with PBS or eMSC-EV-enriched preparations (red rectangle: high-power field; scale bar = 100 μm) and quantification of α-SMA density. **D**, **E** Relative mRNA levels of collagen I and III in wound tissue after treatment. The data are shown as mean ± SEM (***p < 0.001). eMSC-EVs in the figures represent eMSC-EV-enriched preparations

Excessive activation of fibroblasts and overproduction of extracellular matrix (ECM) components contribute to pathological scar formation. Given the observed inhibitory effects of eMSC-EV-enriched preparations on fibroblast activity, we investigated whether this treatment also affects fibroblast contractility, as indicated by α-smooth muscle actin (α-SMA) expression and ECM-related markers. In vitro analysis demonstrated that treatment with eMSC-EV-enriched preparations resulted in a dose-dependent reduction of α-SMA protein expression (Fig. 5A). Consistently, immunohistochemical analysis of wound tissues revealed significantly lower α-SMA expression in the eMSC-EV-enriched preparations-treated group compared to the PBS control (Fig. 5B, C; *p* < 0.001). To further evaluate ECM-related changes, we quantified the mRNA expression levels of collagen type I and collagen type III. As shown in Fig. 5D and E, both collagen I and collagen III transcripts were significantly decreased in the eMSC-EV-enriched preparations-treated group relative to the PBS control (*p* < 0.001). Collectively, these results indicate that eMSC-EV-enriched preparations reduce fibroblast contractility-associated α-SMA expression and downregulate collagen I and III transcripts, suggesting a potential modulatory effect on fibroblast activation and ECM production.

### miR-125b-5p is enriched in eMSC-EV-enriched preparations and is associated with Smad2 expression

**Fig. 6:**
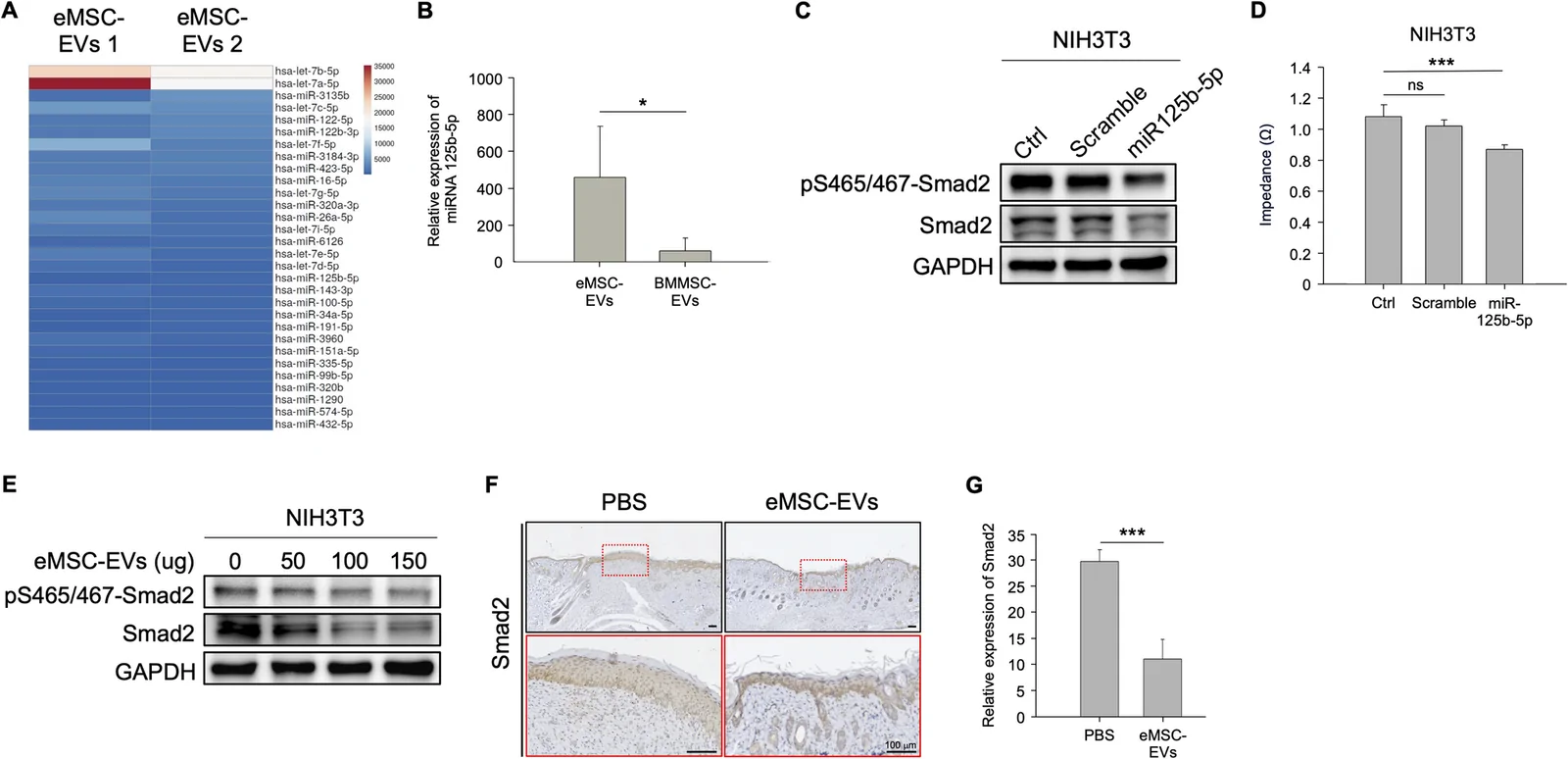
miR-125b-5p is enriched in eMSC-EV-enriched preparations and associated with Smad2 expression. **A** Heatmap of miRNA results for eMSC-EV-enriched preparations. **B** qPCR analysis of miR-125b-5p expression in eMSC-EV-enriched preparations compared to BMMSC-EV-enriched preparations. **C** Immunoblot analysis of Smad2 and p-Smad2 expression in fibroblasts transfected with non-targeting negative control, scramble control, or miR-125b-5p (Full-length blots are presented in Supplementary Fig. 3). **D** Real-time analysis of fibroblast proliferation following transfection with non-targeting negative control, scramble control, or miR-125b-5p using the MaestroZ real-time cell analyzer. **E** Dose-dependent effects of eMSC-EV-enriched preparations on Smad2 and p-Smad2 protein expression in fibroblasts (Full-length blots are presented in Supplementary Fig. 4). **F**, **G** Immunohistochemical staining and quantification of Smad2 expression in wound tissue treated with PBS or eMSC-EV-enriched preparations (red rectangle: high-power field; scale bar = 100 μm). *n* = 5. The data are shown as mean ± SEM (*p < 0.05, ***p < 0.001, ns: no difference). eMSC-EVs and BMMSC-EVs in the figures represent eMSC- and BMMSC-EV-enriched preparations, respectively

To investigate mechanisms underlying the anti-fibrotic effects of eMSC-EV-enriched preparations, we analyzed the microRNA (miRNA) cargo of the preparations using miRNA sequencing. Heatmap analysis revealed that miR-125b-5p was among the top 30 most highly expressed miRNAs and was predicted to target the fibrosis-related gene *Smad2* (Fig. 6A). qPCR validation confirmed that miR-125b-5p expression was significantly higher in eMSC-EV-enriched preparations compared with BMMSC-EV-enriched preparations (Fig. 6B, *p* < 0.05). To assess whether miR-125b-5p influences Smad2 expression, fibroblasts were transfected with miR-125b-5p, a scramble control, or a non-targeting negative control. Transfection of miR-125b-5p reduced total Smad2 and phosphorylated Smad2 (p-Smad2) protein levels, whereas the scramble control showed no significant effect (Fig. 6C). In addition, miR-125b-5p transfection was associated with reduced fibroblast proliferation (Fig. 6D, *p* < 0.001). We further examined whether eMSC-EV-enriched preparations affect Smad2 signaling. Immunoblot analysis demonstrated a dose-dependent reduction of Smad2 and p-Smad2 expression in fibroblasts treated with eMSC-EV-enriched preparations (Fig. 6E). Consistently, Smad2 expression was significantly reduced in wound tissues following treatment with eMSC-EV-enriched preparations in vivo (Fig. 6F, G, *p* < 0.001). Collectively, these findings indicate that miR-125b-5p is enriched in eMSC-EV-enriched preparations and is associated with the modulation of Smad2 signaling, suggesting its possible contribution to the observed therapeutic effects.

### eMSC-EV-enriched preparations attenuated fibrosis in a bleomycin-induced co-administration model

**Fig. 7:**
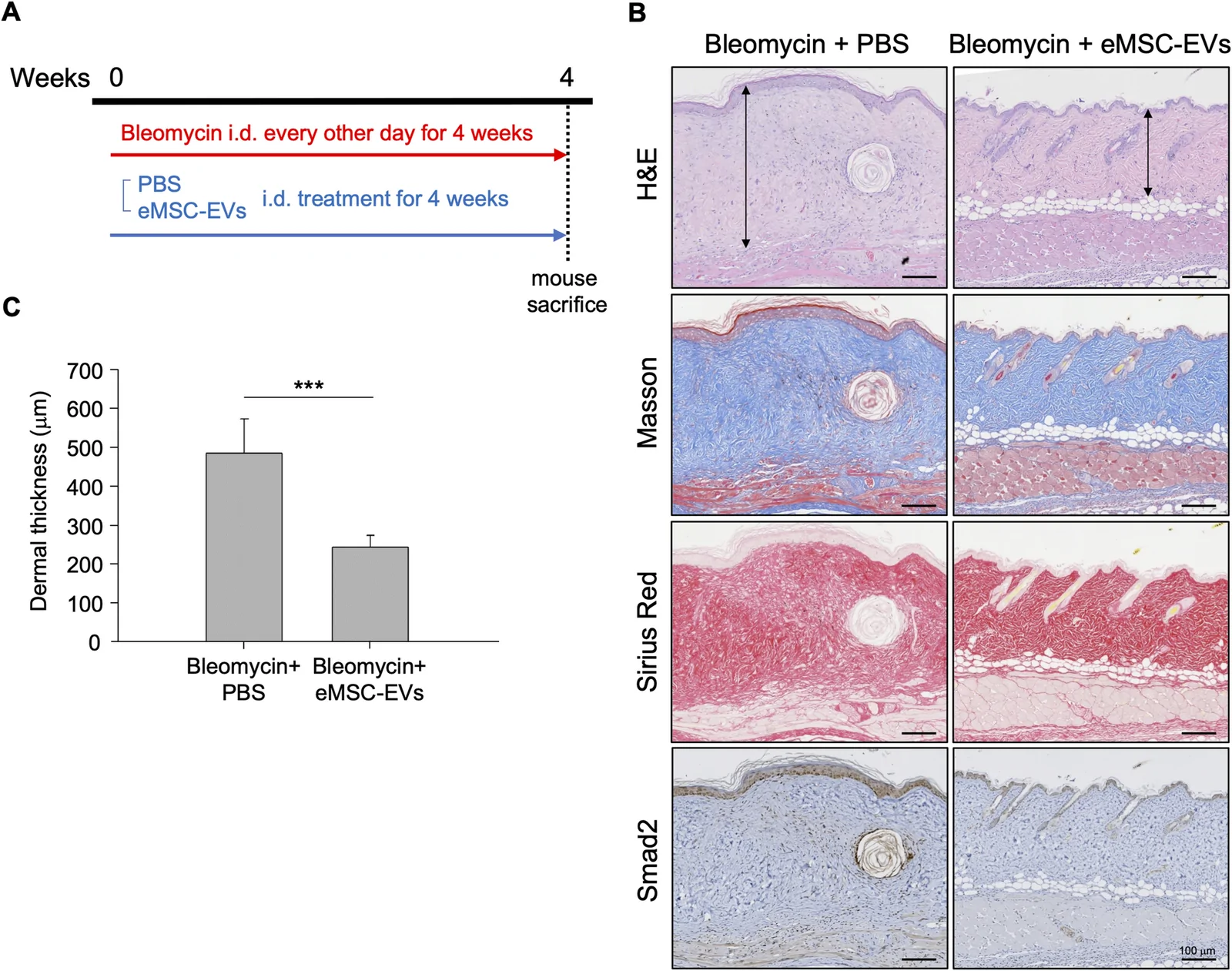
eMSC-EV-enriched preparations attenuate fibrosis in a bleomycin-induced mouse model. **A** Schematic diagram illustrating the experimental design. **B** Representative histological images of skin sections stained with H&E, Masson’s trichrome, Sirius Red, and immunohistochemistry for Smad2 (black line: dermal area; scale bar = 100 μm). **C** Quantification of dermal thickness. *n* = 5. The data are shown as mean ± SEM (***p < 0.001). eMSC-EVs in the figures represent eMSC-EV-enriched preparations

Given that miR-125b-5p in eMSC-EV-enriched preparations suppresses Smad2 expression in vitro, we next evaluated their anti-fibrotic effects in a bleomycin-induced dermal fibrosis model. C57BL/6 mice received intradermal bleomycin injections every other day for four weeks, with concurrent administration of PBS or eMSC-EV-enriched preparations at the same site and schedule (Fig. 7A). Mice treated with eMSC-EV-enriched preparations exhibited reduced dermal thickness compared with the PBS controls. Masson’s trichrome and Sirius Red staining indicated decreased total collagen deposition and a more organized collagen fiber arrangement in the eMSC-EV-enriched preparations group. Immunohistochemical analysis demonstrated lower Smad2 expression in the eMSC-EV-enriched preparations-treated tissues relative to the PBS controls (Fig. 7B, C; *p* < 0.001). Collectively, these results suggest that co-administration of eMSC-EV-enriched preparations attenuates fibrosis development in vivo.

## Discussion

In this study, we provide evidence that extracellular vesicle-enriched preparations derived from human endometrial mesenchymal stem cells (eMSC-EV-enriched preparations) exert anti-fibrotic effects and support tissue remodeling. These effects are mediated through the modulation of fibroblast activity and key molecular pathways involved in fibrosis. Although wound healing is a tightly regulated process, it often results in fibrotic scarring, even under optimal conditions [44, 45]. Improving the quality of wound repair to achieve more regenerative outcomes remains a major challenge in regenerative medicine. The human endometrium provides a unique model of scar-free repair due to its cyclic regeneration following menstruation [46, 47]. eMSCs, residing in the basal layer, contribute to this regenerative capacity and represent a promising source for the development of novel regenerative therapies [48].

Fibroblasts play a central role in wound healing; however, their dysregulation leads to excessive ECM deposition and scar formation [49–52]. Current anti-fibrotic treatments are often limited by side effects and high recurrence rates [53–56]. In the present study, eMSC-EV-enriched preparations inhibited fibroblast proliferation and migration in vitro, and reduced invasion in a Matrigel-based assay, which serves as a supportive proxy for fibroblast behavior under matrix-like conditions. While this in vitro assay does not directly model fibrosis in vivo, it provides useful information on fibroblast functional responses relevant to tissue remodeling.

The human endometrium’s capacity for cyclic, scar-free regeneration may endow eMSC-EV-enriched preparations with distinct anti-fibrotic properties [35]. miRNA profiling identified miR-125b-5p as one of the most enriched miRNAs in eMSC-EV-enriched preparations, consistent with previous reports implicating members of the miR-125 family in fibrosis regulation [57]. In vitro transfection experiments demonstrated that miR-125b-5p can reduce Smad2 and p-Smad2 expression. In parallel, treatment with eMSC-EV-enriched preparations decreased Smad2 signaling both in vitro and in vivo, suggesting that miR-125b-5p may participate in the regulation of Smad2-associated pathways during treatment. However, the current data indicate an association rather than definitive proof of a required mechanistic pathway. Given the complex composition of the EV-enriched preparations’ cargo, multiple miRNAs and other bioactive molecules likely act cooperatively to mediate the overall anti-fibrotic effects and tissue remodeling observed. Therefore, miR-125b-5p should be considered a potential contributor within a broader regulatory network rather than the sole mediator of eMSC-EV-enriched preparations’ function.

In our study, eMSC-EV-enriched preparations exhibited greater anti-fibrotic and tissue remodeling effects than BMMSC-EV-enriched preparations. Similarly, adipose-derived stem cell extracellular vesicles (ASC-EVs) have been extensively explored for wound healing, largely attributed to their pro-angiogenic and immunomodulatory functions mediated by microRNA cargo [58–60]. eMSC-EV-enriched preparations exhibit anti-fibrotic effects that may involve miR-125b-5p/Smad2 signaling, potentially differing from ASC-EVs in miRNA composition (e.g., miR-146a-5p/JAZF1 and miR-10b/CDK6). Given their distinct tissue origins, ASC-EVs and eMSC-EV-enriched preparations may carry unique miRNA profiles that differentially regulate molecular pathways involved in tissue repair. These variations in miRNA cargo could underlie the observed differences in their therapeutic effects, suggesting that tissue-specific EV signatures may contribute to differences in fibrotic and structural recovery responses.

Beyond cellular therapies, eMSC-EV-enriched preparations may offer advantages over conventional wound dressings, which primarily provide passive protection and moisture retention. Unlike these inert materials, eMSC-EV-enriched preparations actively modulate the wound microenvironment through molecular signaling, and have been reported to promote fibrosis resolution, appendage regeneration, and neovascularization [61]. In our full-thickness wound model, eMSC-EV-enriched preparations enhanced angiogenesis and cell proliferation (CD31^+^, Ki67^+^), and improved collagen organization compared with PBS, BMMSC-EV-enriched preparations, or topical antibiotics. However, murine dorsal wound healing is largely driven by contraction rather than re-epithelialization, and splinting was not applied in this study; therefore, wound area reduction may partially reflect contraction. Nevertheless, histological analyses of collagen organization, appendage-like structures, angiogenesis, and proliferation provide complementary evidence supporting the beneficial effects of eMSC-EV-enriched preparations on tissue remodeling. Moreover, unlike standard treatments that may impair hair follicle formation [62], eMSC-EV-enriched preparations were associated with the presence of appendage-like structures in regenerated tissue. Collectively, these findings suggest that eMSC-EV-enriched preparations represent a biologically active therapeutic approach with potential to support multiple aspects of tissue repair.

Fibroblasts are key regulators of tissue repair during wound healing, but dysregulated fibroblast activity can result in excessive ECM deposition and fibrosis [63, 64]. Increased expression of α-smooth muscle actin (α-SMA) and ECM proteins such as collagen I and III is commonly associated with pro-fibrotic responses and scar formation [2, 3, 65, 66]. In our study, treatment with eMSC-EV-enriched preparations reduced the expression of α-SMA, collagen I, and collagen III. These findings suggest that eMSC-EV-enriched preparations may modulate fibrotic responses during wound repair. While these results do not directly define specific differentiation processes, they support a role for eMSC-EV-enriched preparations in influencing ECM remodeling and scar-related responses in vivo.

To enable clinical application, the production of eMSC-EV-enriched preparations must meet requirements for yield, scalability, and regulatory compliance. Emerging bioengineering approaches, such as electrical stimulation, have been shown to enhance MSC secretory activity, thereby improving EV yield and potency [67]. These strategies may also be applied to eMSC-EV-enriched preparations’ production to further optimize both output and functional efficacy. For scalable manufacturing, tangential flow filtration (TFF) offers a GMP-compliant alternative to ultracentrifugation, enabling efficient EV isolation while preserving vesicle integrity [68]. To fulfill regulatory standards, eMSC-EV-enriched preparations’ production must incorporate stringent quality control measures, including assessments of sterility, identity, and potency. Given donor variability, the adoption of standardized potency assays, such as those developed for ASC-derived products, is critical for ensuring product consistency and clinical applicability [69]. Collectively, these approaches provide a feasible framework for advancing eMSC-EV-enriched preparations toward clinical-grade production.

In summary, eMSC-EV-enriched preparations support tissue repair and modulate fibrotic responses, potentially through the miR-125b-5p/Smad2 axis. By delivering miR-125b-5p to influence Smad2 signaling, eMSC-EV-enriched preparations may contribute to improved wound healing, representing a promising cell-free approach for skin repair. These findings provide a rationale for further investigation of eMSC-EV-enriched preparations in fibrotic skin disorders and for advancing their clinical development.

## Conclusions

Taken together, this study provides evidence that eMSC-EV-enriched preparations support cutaneous wound healing. In vivo, wounds treated with eMSC-EV-enriched preparations showed improved closure, increased appendage-like structures, and a more organized collagen matrix, with reduced fibrotic deposition compared with the controls. These effects were accompanied by enhanced angiogenesis and cell proliferation, processes relevant to tissue remodeling. In vitro, eMSC-EV-enriched preparations modulated fibroblast behavior, reducing proliferation, migration, and invasive responses, which may contribute to limiting excessive extracellular matrix deposition during wound repair. Mechanistically, these effects may involve the modulation of Smad2 signaling by miR-125b-5p, with eMSC-EV-enriched preparations’ treatment being associated with reduced expression of α-SMA and other fibrosis-related markers. Collectively, these findings highlight the potential of eMSC-EV-enriched preparations as a cell-free approach to improve tissue remodeling and attenuate fibrosis, supporting further investigation toward clinical translation in regenerative medicine.

## Supplementary Information

Below is the link to the electronic supplementary material.

Supplementary Material 1

## Data Availability

No datasets were generated or analysed during the current study.

## Abbreviations

α-SMA: α-Smooth muscle actin
ASC-EVs: Adipose-derived stem cell extracellular vesicles
BMMSCs: Bone marrow-derived mesenchymal stem cells
BSA: Bovine serum albumin
DAB: 3,3′-Diaminobenzidine
DAPI: 4′,6-Diamidino-2-phenylindole
DMEM: Dulbecco’s modified Eagle medium
DMEM/F-12: Dulbecco’s modified Eagle medium/nutrient mixture F-12
ECL: Enhanced chemiluminescence
ECM: Extracellular matrix
eMSCs: Endometrial mesenchymal stem cells
FBS: Fetal bovine serum
GAPDH: Glyceraldehyde-3-phosphate dehydrogenase
H&E: Hematoxylin and eosin
HBSS: Hank’s balanced salt solution
HRP: Horseradish peroxidase
IHC: Immunohistochemistry
miRNA: MicroRNA
miR-125b-5p: MicroRNA-125b-5p
MSC-EVs: MSC-derived extracellular vesicles
NTA: Nanoparticle tracking analysis
PBS: Phosphate-buffered saline
Pen/Strep: Penicillin-streptomycin
PVDF: Polyvinylidene difluoride
Smad2: Mothers against decapentaplegic homolog 2
TBST: Tris-buffered saline with Tween-20
TEM: Transmission electron microscopy
TFF: Tangential flow filtration
