# Supplementary material for: Regenerative potential of extracellular vesicles from endometrial mesenchymal stem cells for modulating fibrosis and wound healing
Source: Stem Cell Res Ther. 2026 Jul 18;17:368. doi: 10.1186/s13287-026-05189-w (PMC13670864; doi:10.1186/s13287-026-05189-w)
Supplement: Supplementary file 1 [file 13287_2026_5189_MOESM1_ESM.docx]

Supporting information for

**Regenerative potential of extracellular vesicles from endometrial mesenchymal stem cells for modulating fibrosis and wound healing**

Fang-Yu Lin^1,2^, Xiao-Tong Tan^2^, Chih-Ming Pan^2^, Chien-Chu Huang^5,6,7^, Wan-Ling Chiang^2^, Che-Kai Chang^2^, Wu-Chou Lin^5,6^, Chiung-Yi Yeh^1,5,6^, Yu-Chuan Lin^2^, Ting-Hsun Lin^2^, Der-Yang Cho^1,2,3,4^, and Shao-Chih Chiu^1,2,3^

*^1^Graduate Institute of Biomedical Sciences, China Medical University, Taichung, Taiwan, R.O.C.;
^2^Translational Cell Therapy Center, China Medical University Hospital, Taichung, Taiwan, R.O.C.;
^3^Drug Development Center, China Medical University, Taichung, Taiwan, R.O.C.;
^4^Department of Neurosurgery, China Medical University Hospital, Taichung, Taiwan, R.O.C.;
^5^Department of Obstetrics and Gynecology, China Medical University Hospital, Taichung, Taiwan, R.O.C.
^6^Department of Medicine, School of Medicine, China Medical University Hospital, Taichung, Taiwan, R.O.C.
^7^Center for immune-Oncology, National Cancer Institute, National Institutes of Health, Bethesda, MD, U.S.A.*

Fang-Yu Lin, Xiao-Tong Tan and Chih-Ming Pan contributed equally to this work

Correspondence to:
Shao-Chih Chiu ([s-cchiu@outlook.com](mailto:s-cchiu@outlook.com)) and Der-Yang Cho ([dycho@tool.caaumed.org.tw](mailto:dycho@tool.caaumed.org.tw))

**Supporting figures**

**Supplementary figure 1.** Full-length blots.
Full length blots of Figure 1F.

**Supplementary figure 2.** Full-length blots.
Full length blots of Figure 5A.

**Supplementary figure 3.** Full-length blots.
Full length blots of Figure 6C.

**Supplementary figure 4.** Full-length blots.
Full length blots of Figure 6E.
